# Diurnal Variation of Tear and Meibum Lipids in Healthy Subjects

**DOI:** 10.1007/s44402-026-00112-5

**Published:** 2026-05-27

**Authors:** Mukesh Kumar, Ankit Raj, Ajay Kumar Vijay, Srikanth Dumpati, Simin Masoudi, Mark Willcox

**Affiliations:** 1 School of Optometry and Vision Science, University of New South Wales, Sydney, New South Wales Australia; 2 LV Prasad Eye Institute, Hyderabad, India; 3 Centre for Vision Research, Westmead Institute for Medical Research, The University of Sydney, Sydney, New South Wales Australia

**Keywords:** Meibomian glands, Meibum, Meibum composition, Meibum lipids, Tears

## Abstract

**Purpose:**

To determine whether the lipid composition of tears and meibum demonstrates diurnal variation in healthy individuals, with emphasis on the relative distribution of lipid classes.

**Methods:**

Fifteen non–contact lens wearers (10 females, 5 males; mean age 29.5 ± 11.6 years) provided morning (09:00–10:00 h) and evening (16:00–17:00 h) tear and meibum samples. Tears were collected with Schirmer strips without anaesthesia, and meibum was expressed from the lower lid margin using a Korb expressor and collected with a sterile spatula under slit-lamp guidance. Lipids were extracted in chloroform and analysed by mass spectrometry. Relative molar proportions of wax esters (WE), cholesteryl esters (CE), triglycerides (TG) and polar lipid classes were quantified.

**Results:**

Non-polar lipids comprised most tear lipids in the morning (98.8%) and evening (99.0%), with WE being most abundant, followed by CE and TG. No significant diurnal differences were observed in tear lipid class proportions. Meibum lipid composition was also dominated by non-polar species (99.4% morning; 99.2% evening), with WE as the principal component. TG levels were higher in evening meibum, while diacylglycerol and phosphatidylserine were significantly elevated among polar lipids (*p* = 0.04 and *p* = 0.03, respectively).

**Conclusion:**

The overall distribution of major non-polar lipids in tears and meibum remains stable throughout the day in healthy individuals. Minor changes in specific lipid classes suggest potential molecular-level modulation, the functional relevance of which warrants further investigation.

Key Points

- This study investigated whether the lipid composition of tears and meibum changes between morning and evening in healthy individuals.
- The overall distribution of major tear and meibum lipids remained stable throughout the day, with non-polar lipids and wax esters being the dominant lipid classes.
- Minor changes in some meibum lipids suggest possible diurnal molecular variation.

## Introduction

Dry eye is a multifactorial, symptomatic disease characterised by a loss of homeostasis of the tear film and/or ocular surface, in which tear film instability and hyperosmolarity, ocular surface inflammation and damage and neurosensory abnormalities are aetiological factors [1, 2]. The purpose is to keep the cornea covered with a thin coating of mostly aqueous tears that provide lubrication and protection to the ocular surface. The chemical composition of the tear fluid is closely linked with the physical properties of the tear film. Clinical issues such as dry eye can develop as a consequence of the tear film’s inadequacy to provide adequate lubrication and protection [3].

The tear lipid layer is a thin coating that spreads across the aqueous layer of the tear film with each blink, contributing to the formation of a smooth optical surface at the front of the eye [4, 5] and reducing tear film evaporation [6]. Most of the lipids in the tear film are produced by the meibomian glands [7]. Tear film lipids lower the surface tension of the aqueous layer, allowing it to spread more smoothly across the eye with each blink [8]. This prevents the tear film from collapsing onto the ocular surface and helps stabilise the air/tear interface [8, 9]. A thicker tear film lipid layer has been associated with significantly slower evaporation flux and delayed tear breakup [10]. While meibum from both healthy individuals and those with meibomian gland dysfunction (MGD) reduces water evaporation by approximately 26%, measurements of evaporation from the tear menisci revealed that meibum from individuals with MGD resulted in a 25% higher evaporation rate compared with normal subjects [10]. Dursch et al. reported that adequate lipid layer coverage can reduce tear film evaporation by 50–95%, relative to the evaporation rate of pure water [11].

Cholesteryl esters (CEs), wax esters (WEs) and triacylglycerols (TAGs) are the most prevalent non-polar lipids in human tears [7]. WEs are highly diverse molecules, composed of very long-chain fatty acids and fatty alcohols, containing zero to four double bonds [12]. *In vitro* studies showed that WEs play a role in reducing tear evaporation [13]. The relative concentration of WE is lower in the tears of patients with MGD, compared with healthy individuals [14]. CEs are formed by esterifying a fatty acid chain (such as C16 or C20) to cholesterol, while TAGs are derived from the esterification of glycerol with three fatty acids. A link has been observed between the fatty acid composition of TAGs and chronic blepharitis [15]. Additionally, higher levels of CE have been found in the meibum of patients with moderate dry eye, compared with those having mild dry eye [16]. The CE to WE ratio decreases in patients with MGD [14, 17, 18]. A balanced mixture of WE and CE in the tear fluid lipid layer may be essential for disrupting the ordered packing of pure lipid species, promoting better lipid spreading [19] and a more stable tear film [20]. Thinner lipid layers have been linked with a reduction in the levels of non-polar lipids, such as CE and an increase in polar lipids, like ceramides (CERs) [21].

Lipids are fundamental to maintaining tear film stability and ocular surface homeostasis [22]. Alterations in the composition of TAGs and phosphatidylcholines (PCs) have been implicated in dry eye disease (DED), underscoring their contribution to tear film dynamics and evaporative resistance [23]. CERs are essential for preserving meibomian gland structure [24] and exert potent anti-inflammatory effects [24], whereas elevated sphingomyelin (SM) levels have been associated with oxidative stress and cell damage on the ocular surface [25]. Phosphatidylethanolamine (PE) contributes to membrane organisation and lipoprotein stability [26]. Characterising temporal fluctuations in these lipid species may provide novel insights into tear film regulation and the pathophysiology of ocular surface disorders [26].

It is well established that ocular surface comfort declines from morning to evening, particularly during activities such as prolonged computer use, exposure to air conditioning and contact lens wear [27, 28]. However, to date, no significant correlations have been found between biochemical changes to most of the proteins, lipids and inflammatory mediators in tears and the increase in ocular discomfort at the end of the day [29]. The levels of certain TAG and WE species change from morning to evening, but whether these changes are correlated with ocular comfort [27] or whether there are also diurnal changes in lipids that may correlate with ocular comfort changes has not been investigated. Therefore, the aim of this study was to assess the levels of meibum and tear film lipids throughout the day. It was hypothesised that the levels of different lipid classes present in tears and meibum would fluctuate across the day, indicating a diurnal regulation of ocular surface lipid composition.

## Methods

### Study Design

This prospective study was carried out at the School of Optometry and Vision Science, University of New South Wales (UNSW), Sydney, Australia. The research complied with the principles of the Declaration of Helsinki and received approval from the Human Research Ethics Committee at UNSW (HC210010). Informed consent was obtained from all participants prior to their enrolment in the study.

### Participants

Fifteen healthy participants aged 18 years and older were recruited for the study. Individuals who had received intensive meibomian gland therapies such as moist heat compresses, thermal pulsation treatment or intense pulsed light therapy within the preceding 12 months were excluded from participation [30]. Other exclusion criteria included active eye infections, inflammation, allergies, recent eye injury or surgery within the past 6 months, systemic diseases affecting ocular physiology and the use of corticosteroids, immunosuppressants or antihistamines prior to enrolment [30, 31]. Pregnant or breastfeeding women, as well as individuals who wore contact lenses, were also excluded from participation [32].

### Study Procedures

Tear and meibum samples were collected during two study sessions conducted between 09:00–10:00 h (morning) and 16:00–17:00 h (evening) to evaluate potential diurnal variations. These time points were chosen to capture lipid composition shortly after the start of daily activities and toward the end of the day, when tear film stability and symptoms are often poorer and ocular surface stress is typically greater [33]. Sampling at only two clinically anchored time points also limited participant burden and avoided excessive reflex tearing associated with more frequent collections [34].

**Fig. 1:**
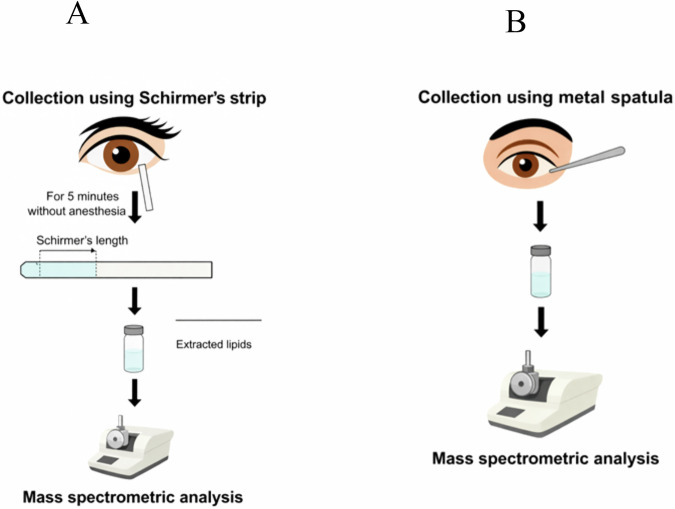
Representative schematic illustrations showing tear and meibum collection methods. **A** Tear collection using Schirmer’s strip for 5 min without anaesthesia, followed by lipid extraction and mass spectrometric analysis. **B** Meibum collection using a metal spatula, with subsequent lipid extraction and mass spectrometric analysis.

During each session, tear samples were collected using Schirmer’s strips (Tearflo; HUB Pharmaceuticals LLC, hubrx.com) placed in the temporal one-third of the lower conjunctival fornix of each eye without topical anaesthesia. Participants were instructed to gently close their eyes during the 5-min collection period, and the strips were then transferred into sterile glass vials and immediately stored at −80 °C until lipid extraction and analysis (Fig. 1A) [35]. Following tear collection, a 10-min washout period was allowed to facilitate stabilisation of the tear film and minimise the influence of reflex tearing on subsequent procedures.

Meibum samples were collected from the lower eyelid of both eyes. The lower eyelid was gently everted, and meibum was expressed using a Korb expressor (TearScience, jnjvisionpro.com) positioned just below the eyelash line for 10 s to ensure adequate expression [36]. The expressed meibum was carefully collected from the lid margin using a sterile metal spatula and transferred into a glass vial (Fig. 1B). All collections were performed while viewing through a slit lamp biomicroscope (Carl Zeiss Pty Ltd, zeiss.com) by the same investigator to minimise inter-observer variability. The order of procedures (tear sampling followed by meibum collection) was kept constant across all participants and sessions. Potential external factors influencing tear film stability and meibum secretion were controlled. All measurements were obtained at standardised time points under comparable environmental conditions, and participants were instructed to avoid ocular cosmetics, facial products and prolonged screen exposure prior to each visit [28]. All samples (morning and evening) were processed and analysed within a single liquid chromatography-mass spectrometry run to minimise variability. The injection sequence was arranged to reduce potential systematic bias between time points. In addition, pooled quality control samples, prepared by combining aliquots from all samples, were injected at regular intervals throughout the analytical sequence to monitor instrument stability and analytical performance.

Samples were dissolved in 300 μL chloroform immediately after collection and stored at −80 °C until lipid analysis [36]. The eyelid margin was not cleaned before collection, as previous studies have reported that cleaning does not alter meibum composition [37]. During collection, the eyelid was gently turned away from the ocular surface to avoid tear contamination [38].

### Quantification of Total Lipids

#### Chemicals

The chemicals used were largely as described previously [27]. Methanol, chloroform, methyl tert-butyl ether (MTBE), ammonium acetate (suitable for liquid chromatography-mass spectrometry) and analytical-grade butylated hydroxytoluene (BHT) were sourced from Sigma-Aldrich (sigmaaldrich.com). The phospholipid standards were obtained from Avanti Polar Lipids (avantiresearch.com). Standards for WE and CE were procured from Nu-Chek Prep (nu-chekprep.com), and TAG standards were supplied by CDN Isotopes (lgcstandards.com).

### Lipid Extraction from Meibum Samples

The extraction of meibum lipids followed a previously established method [7, 39]. In brief, lipid standards were dissolved in pure methanol to prepare a standard solution, with the molar concentrations of the lipid’s internal standard solution (Table 1). Fifty microliters of this internal standard solution were added to each dry meibum sample. A mixture of MTBE and methanol (200 μL (10:3 vol/vol; containing 0.01% BHT)) was added, and the samples mixed using an orbital shaker for 30 min at room temperature.

**Table 1:** Molar concentrations of internal standards in the stock solution.

Name of lipids	Concentrations picomolar (pM) in the internal standard
Cholesteryl tridecanoate (CE)13:0	320
Ceramide (Cer) 17:0	40
Diacylglycerols (DAGs) 17:0	40
Free cholesterol (FC-D7)	680
Lysophosphodylcholine (LPC) 17:0	40
Phosphatidylethanolamine (PE) 17:0/17:0	40
Triacylglycerols (TAG-D5) 48:0	40
Sphingomyelin (SM) 18:0/12:0	40
Palmityl palmitoleate (WE) 16:0/16:0	80
Phosphatidylserine (PS) 17:0/17:0	40
Phosphatidylcholine (PC) 19:0/19:0	40
Lysophosphatidylethanolamine (LPE) 14:0	40

The internal standards in these experiments had similar chemical structures and relative concentrations of each lipid class in meibum samples [27]. The internal standards accounted for both random and systematic uncertainties arising from sample preparation or instrument fluctuations. A biphasic lipid extraction was performed by adding 50 µL of 0.15 M ammonium acetate to the MTBE solution, followed by vortexing for >30 s to ensure thorough mixing. The samples were centrifuged for 5 min at 2000 × *g* to achieve phase separation, and the upper MTBE layer was collected for subsequent processing. The MTBE (top) phase was carefully transferred to a new vial without disturbing the aqueous layer and dried under a gentle stream of nitrogen at 37 °C. The dried lipid extract was reconstituted in 100 µL of 2:1 methanol/chloroform containing 5 mM ammonium acetate and vortex-mixed to ensure complete dissolution. Different internal standard concentrations were selected for each lipid class to match the expected endogenous abundance of neutral and polar lipids, thereby maintaining detector linearity and avoiding signal saturation. Class-specific levels were further optimised to account for differences in ionisation efficiency and matrix effects between non-polar and polar species, ensuring accurate recovery and precision across the dynamic range [40, 41]. Samples were then stored at −80 °C until mass spectrometry analysis.

### Mass Spectrometry

Mass spectrometry was conducted following a previously established protocol [42]. A 22-µl sample of the extracts was injected into a Q-Exactive Plus Mass Spectrometer (ThermoFisher Scientific, thermofisher.com) connected to a U3000 ultra-pressure liquid chromatography system (ThermoFisher Scientific, thermofisher.com). Data were acquired in positive electrospray ionisation (ESI⁺) mode. The solvents used were solvent A, a 6:4 acetonitrile mixture and solvent B, a 1:9 acetonitrile mixture, both containing 10 mM ammonium formate and 0.1% formic acid. Lipid separation was performed using a method described earlier [43], involving a 30-min gradient from 30 to 100% of solvent B, which allowed lipids to elute in order of hydrophobicity. Chromatography was carried out at 60 °C on a Waters CSH C18 UHPLC column (2.1 × 100 mm, 1.8 µm) with a VanGuard pre-column (Waters Corporation, waters.com). The column eluate was directed into the mass spectrometer’s ESI source, using a heated ESI-I probe, with source parameters optimised for various lipid standards before analysis.

For lipid identification, Lipid Search software version 4.1 (ThermoFisher Scientific, thermofisher.com) was employed. The software utilised both a parent search mode, based on the accurate mass of precursor ions and a product search mode, which considered both the precursor ion mass and the MS2 spectral pattern. The precursor and product mass tolerances were set to a 5-parts per million window. Precursor ion relative intensity threshold was set at 1% and the product ion threshold at 5%, with the m-score threshold at 2.0 [44]. The data were exported to a Microsoft Excel (Microsoft.com) spreadsheet for manual processing and statistical analysis. The raw abundances (peak areas) were normalised by dividing each peak area by the raw abundance of the corresponding internal standard for that lipid class.

### Data Analysis

Statistical analyses were performed using IBM SPSS (version 29.0; ibm.com) and GraphPad Prism (version 10.0; graphpad.com). Data are presented as mean ± SD. Normality was assessed using the Shapiro–Wilk test. Paired comparisons between morning and evening samples were conducted using paired *t*-tests or Wilcoxon signed-rank tests, as appropriate. Analyses evaluated differences in the relative proportions of lipid classes. Given multiple comparisons, results should be interpreted with caution; unadjusted *p* values are reported. A two-tailed *p* < 0.05 was considered statistically significant. The sample size was calculated a priori assuming a moderate effect size (Cohen’s *d* ≈ 0.60), with an alpha of 0.05 and 80% power, based on variability reported in previous tear and meibum lipidomic studies [7, 45]. The analysis indicated that a minimum of 12 participants was required. To account for potential data loss and ensure consistency with prior studies, 15 participants were enroled.

### Ethics Approval and Consent to Participate

Ethical approval was obtained prior to study commencement, and the study adhered to the tenets of the Declaration of Helsinki. Written informed consent was obtained from all participants before participation.

## Results

**Fig. 2:**
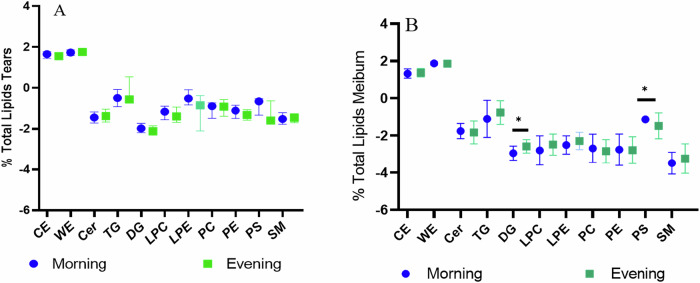
Mole percentage of total lipids in morning and evening samples.**A** Tear lipid mole percentage (log-transformed). **B** Meibum lipid mole percentage (log-transformed). CE cholesterol esters, WE wax esters, Cer ceramide, TG triacylglycerides, DG diacylglycerols, LPC lysophosphatidylcholine, LPE Lysophosphatidylethanolamine, PC phosphatidylcholine, PE phosphatidylethanolamine, PS phosphatidylserine, SM sphingomyelin. Quantitative comparisons of lipid profiles in the morning and evening samples are presented as median mole per cent and interquartile range. Asterisks denote significant differences based on log-transformed data (*P < 0.05).

Fifteen participants (10 women and 5 men) with an average age of 29.53 ± 11.57 years completed the study. The mole percentages of polar and non-polar lipids in tears and meibum are summarised in Table 2. In tears, non-polar lipids comprised the majority of the composition, accounting for 98.8% in the morning and 99.0% in the evening. WEs were the most abundant class (55.6 ± 22.6 morning vs. 52.6 ± 22.7% evening), followed by CEs (38.4 ± 24.1 vs. 37.1 ± 20.1%) and TGs (3.9 ± 12.3 vs. 8.0 ± 18.1%). Polar lipids made up 1.2% and 1.0% of the tear lipid profile in the morning and evening, respectively, with PC being predominant. No significant differences were observed in the relative proportions of individual lipid classes between morning and evening tear samples (Fig. 2A).

**Table 2:** Mole percentage of polar and non-polar lipids to total lipids.

	Mole per cent to total lipids	Morning	Evening
Tears	Polar lipids (mole %)	1.2	1.0
	Non-polar lipids (mole %)	98.8	99.0
Meibum	Polar lipids (mole %)	0.6	0.8
	Non-polar lipids (mole %)	99.4	99.2

In meibum, non-polar lipids represented 99.4% of the composition in the morning and 99.2% in the evening. WE were the most abundant class (73.3 vs. 72.8%), followed by CE (25.3 vs. 24.3%), with TG levels higher in the evening (0.72 vs. 1.9%). Polar lipids contributed only 0.6% and 0.8% in morning and evening samples, respectively. Among these, diacylglycerols (DGs) and phosphatidylserine (PS) showed significantly higher levels in the evening (*p* = 0.04 and 0.03, respectively), while the remaining polar lipids (LPC, LPE, PC, PE, SM) showed no significant differences (Fig. 2B).

## Discussion

Disruption of the tear film equilibrium is known to influence the organisation and stability of the lipid layer [46, 47]. The lipid layer of the tear film is primarily composed of non-polar lipids, including WEs and CEs, which together constitute approximately 80–95% of the total lipid content [48, 49]. Smaller but functionally significant amounts of polar lipids, such as phospholipids, contribute to the interfacial stability between the aqueous and lipid phases of the tear film [35, 50].

The present findings demonstrated that the relative abundance of non-polar lipid classes, specifically WE, CE and TAG, remained consistent throughout the day. This stability suggests that the non-polar lipid composition of the tear film is tightly regulated and maintains its structural integrity under physiological conditions. Normalisation of each lipid class to total lipid content minimised the influence of inter-individual variability, which is known to be considerable in tear lipid profiles [45].

A previous study reported that cholesterol levels may decrease and PC and SM concentrations may increase across the day, whereas non-polar lipid levels remain largely unchanged [29]. The present results are consistent with these earlier observations, demonstrating that non-polar lipids modulate the interfacial properties of the tear film lipid layer, preserve its hydrophobicity and attenuate tear film destabilisation in the presence of variable meibomian gland secretion or environmental factors.

In this healthy cohort, although several non-polar lipids demonstrated directional tendencies, statistically significant variation was limited to the DG and PS classes, indicating that only a subset of tear film lipids undergo meaningful temporal modulation. The prominence of DG and PS fluctuations supports the concept that compositional changes, rather than total lipid abundance, may play a greater role in ocular surface physiology, as even small alterations in lipid structure or saturation can have disproportionate effects on tear film performance [16, 51]. Previous lipidomic studies have highlighted that subtle modifications in chain length or degree of unsaturation can markedly affect lipid packing characteristics, spreading dynamics and the resistance of the tear film to evaporative forces [40, 52], and these compositional features should be followed up in subsequent studies.

Non-polar lipids remain crucial for stabilising the superficial tear film. They prevent the collapse of the lipid layer and support the formation of a multilayered surface after each blink [53, 54]. WE enriched with saturated fatty acids have been proposed to promote lipid film cohesion and facilitate re-spreading during blinking [55]. The selective modulation of DG and PS observed in this study suggests that diurnal lipid homeostasis is not uniform across all lipid classes and that the physiological mechanisms regulating biosynthesis, secretion or redistribution affect signalling-related phospholipids and intermediate metabolites. These findings support emerging evidence that lipid variability across a narrow subset of molecules influences tear film behaviour and ocular sensation, independent of gross lipid quantity [16, 51]. Collectively, available lipidomic and functional evidence indicates that diurnal alterations in tear and meibum composition are of limited magnitude. Masoudi et al. [27] reported that approximately 90% of non-polar tear lipid species remained invariant between morning and evening, with detectable diurnal variation restricted to a small subset of low-abundance TAG and WE species. Future work should clarify whether such fluctuations represent adaptive regulatory processes or early biochemical signatures of altered ocular surface function in at-risk populations.

### Limitations

A limitation of this study is the lack of a comprehensive, standardised assessment of DED, including both symptom evaluation and full clinical signs. Although participants with known ocular surface disease were excluded, subclinical DED cannot be entirely ruled out and may have acted as a confounding factor. Randomisation of the sampling sequence was not performed, and tear sampling using Schirmer’s strips was consistently conducted prior to meibum collection; however, a 10-min washout period was implemented to minimise potential procedural interaction, although residual effects cannot be excluded completely. Another limitation is the inclusion of only two diurnal time points (morning and evening), which may not fully capture the complexity of diurnal variations, particularly non-linear or multi-peak patterns.

Future studies should incorporate larger and more heterogeneous cohorts, including individuals with clinically diagnosed DED and habitual contact lens wearers using different lens materials. Such diversity would enable determination of whether alterations in lipid molecular profiles are influenced by ocular surface pathology or by the physicochemical properties of distinct contact lens polymers. In addition to subjective ratings, validated symptom-based questionnaires, including the Contact Lens Dry Eye Questionnaire-8, and Ocular Surface Disease Index, as well as objective clinical assessments of the ocular surface (e.g., non-invasive tear break-up time, tear meniscus height, lipid layer thickness) should be employed to characterise discomfort and its relationship to tear lipid changes. Longitudinal sampling protocols should capture tear and/or meibum at moments when participants actively report discomfort, allowing improved differentiation of lipidomic signatures associated with symptomatic states. Together, these methodological refinements strengthen the ability to link temporal lipid variation with ocular comfort and may clarify how contact lens materials modulate lipid dynamics throughout the day.

## Conclusion

This study shows that the relative distribution of major non-polar lipid classes in tears and meibum remains largely stable between morning and evening in healthy subjects. Minor fluctuations were observed in a specific lipid class, but these changes occurred within low-abundance fractions and did not alter the dominant non-polar profile.

## Data Availability

No datasets were generated or analysed during the current study.
